# ^68^Ga-DOTATOC PET/CT Follow Up after Single or Hypofractionated Gamma Knife ICON Radiosurgery for Meningioma Patients

**DOI:** 10.3390/brainsci11030375

**Published:** 2021-03-15

**Authors:** Fabio Barone, Francesco Inserra, Gianluca Scalia, Massimo Ippolito, Sebastiano Cosentino, Antonio Crea, Maria Gabriella Sabini, Lucia Valastro, Iolanda Valeria Patti, Stefania Mele, Grazia Acquaviva, Alessandra Tocco, Maria Tamburo, Francesca Graziano, Ottavio S. Tomasi, Rosario Maugeri, Gerardo Iacopino, Salvatore Cicero, Lidia Strigari, Giuseppe Emmanuele Umana

**Affiliations:** 1 Gamma-Knife Center, Department of Neurosurgery, Cannizzaro Hospital, 95126 Catania, Italy; fbarone1969@gmail.com (F.B.); inserrafrancesco@hotmail.it (F.I.); 2 Neurosurgery Unit, Highly Specialized Hospital and of National Importance “Garibaldi”, 95122 Catania, Italy; gianluca.scalia@outlook.it (G.S.); fragraziano9@gmail.com (F.G.); 3 Department of Advanced Technologies, Nuclear Medicine and PET Cannizzaro Hospital, 95126 Catania, Italy; ippolitomas@yahoo.it (M.I.); ianocose@hotmail.com (S.C.); 4 Trauma Center, Gamma Knife Center, Department of Neurosurgery, Cannizzaro Hospital, 95126 Catania, Italy; antonio_crea89@virgilio.it (A.C.); cicerosalvatore@yahoo.it (S.C.); 5 Neurosurgery Unit, Department of Clinical-Surgical, Diagnostic and Pediatric Sciences, University of Pavia, 27100 Pavia, Italy; 6 Medical Physics Unit, Cannizzaro Hospital, 95126 Catania, Italy; mgabsabini@gmail.com (M.G.S.); lucia.valastro@gmail.com (L.V.); valeriapatti72@gmail.com (I.V.P.); mele.stefania@gmail.com (S.M.); 7 INFN-Laboratori Nazionali del Sud, Via S. Sofia 62, 95123 Catania, Italy; 8 UOC Radioterapia AOOE Cannizzaro di Catania, 95100 Catania, Italy; gra.acquaviva@libero.it (G.A.); aluccia82@hotmail.it (A.T.); 9 UOC Radiodiagnostica e Radioterapia, AOU Policlinico-VE di Catania, 95125 Catania, Italy; 10 Cannizzaro Hospital, 95100 Catania, Italy; marinellatamburo@virgilio.it; 11 Department of Neurological Surgery Christian Doppler Klinik, Paracelsus Medical University Salzburg, 5020 Salzburg, Austria; ottavio.tomasi@gmail.com; 12 Laboratory for Microsurgical Neuroanatomy Christian Doppler Klinik, 5020 Salzburg, Austria; 13 Neurosurgery Unit, Department of Biomedicine, Neurosciences & Advanced Diagnostics, School of Medicine, University of Palermo, 90127 Palermo, Italy; rosario.maugeri1977@gmail.com (R.M.); gerardo.iacopino@gmail.com (G.I.); 14 Department of Medical Physics, IRCCS Azienda Ospedaliero-Universitaria di Bologna, 40138 Bologna, Italy; lidia.strigari@aosp.bo.it

**Keywords:** ^68^Ga-DOTATOC PET/CT, meningioma, Gamma Knife, radiosurgery, follow up, hypofractionated, gallium

## Abstract

^68^Ga-DOTATOC represents a useful tool in tumor contouring for radiosurgery planning. We present a case series of patients affected by meningiomas on who we performed ^68^Ga-DOTATOC positron emission tomography (PET)/CT pre-operatively, a subgroup of which also underwent a post-operative ^68^Ga-DOTATOC PET/CT to evaluate the standardized uptake value (SUV) modification after Gamma Knife ICON treatment in single or hypofractionated fractions. Twenty patients were enrolled/included in this study: ten females and ten males. The median age was 52 years (range 33–80). The median tumor diameter was 3.68 cm (range 0.12–22.26 cm), and the median pre-radiotherapy maximum SUV value was 11 (range 2.3–92). The average of the relative percentage changes between SUVs at baseline and follow up was −6%, ranging from −41% to 56%. The SUV was reduced in seven out of 12 patients (58%), stable in two out of 12 (17%), and increased in three out of 12 (25%), suggesting a biological response of the tumor to the Gamma Knife treatment in most of the cases. ^68^Ga-DOTATOC-PET represents a valuable tool in assessing the meningioma diagnosis for primary radiosurgery; it is also promising for follow-up assessment.

## 1. Introduction

Meningiomas are common benign, slow-growing, primary intracranial tumors representing up to 36.4% of central nervous system (CNS) neoplasms [1]. Given the progressively increased use of brain MRI for several indications, the diagnosis of asymptomatic meningiomas has increased over the last decades. Their management includes, among others, wait-and-see observation, pharmacological treatment with anti-progesterone, platelet-derived growth factor antagonists, and vascular endothelial-derived growth factor (VEGF) inhibitors. On the other hand, surgical resection is the treatment required for symptomatic meningiomas, usually due to the fact of their growth. Radiosurgery represents an alternative and/or complementary treatment for both small lesions and tumor residues [2,3,4,5,6,7,8,9,10,11,12,13,14,15]. In the literature, Gamma Knife radiosurgery (GKRS) (Elekta AB, Stockholm, Sweden) has been reported to be safe and effective, with a tumor control rate ranging from 88% to 100%, offering reduced morbidity and mortality rates if compared with surgical resection [16]. Somatostatin receptor subtype 2 (SSTR2)-based positron emission tomography (PET), in particular ^68^Ga-DOTATOC, ^68^Ga-DOTATATE, and ^68^Ga-DOTANOC, has been reported as a useful diagnostic tool in meningioma patients, with level 2 evidence in tumor contouring for radiotherapy planning [17]. The radio surgical treatment aims to include all the clonogenic tumor cells of the lesion. The main limitation is represented by its associated microscopic extension, which can be missed with current imaging technologies. This concept is defined as the clinical target volume (CTV), represented by the tumor volume comprehensive of visible gross tumor volume (GTV) and subclinical malignant disease. Experiences with PET/CT or PET/MRI ^68^Ga-DOTATOC, ^68^Ga-DOTATATE, and ^68^Ga-DOTANOC with CyberKnife radiosurgery have been reported in literature, but not with Gamma Knife ICON, in single fraction or hypofractionated treatments. We present a case series of 20 patients affected by meningiomas, on who we performed ^68^Ga-DOTATOC PET/CT pre-operatively, a subgroup of which also underwent a post-operative ^68^Ga-DOTATOC PET/CT to evaluate the standardized uptake value (SUV) modification after Gamma Knife ICON treatment.

## 2. Materials and Methods

We retrospectively analyzed 20 patients treated with Gamma Knife ICON for intracranial meningiomas. The patients underwent preoperative gadolinium-enhanced brain MRIs that showed meningiomas further investigated with ^68^Ga-DOTATOC PET/CT to assess the diagnosis better and make a differential diagnosis. To contour the tumor, all patients underwent volumetric T1-weighted MRIs with gadolinium, in addition to the standard head MRI used to make the diagnosis. After six months, 12 patients underwent post-radiosurgical 68Ga-DOTATOC PET/CT to evaluate possible SUV modifications that could suggest tumor alterations due to the treatment. Data were extracted from the patients’ clinical files, and data on short-term follow up and complications are reported in this article. Overall, this is an observational study.

### 2.1. ^68^Ga-DOTATOC PET/CT

The PETs/CTs was performed using the tracer ^68^Ga-DOTATOC (DOTA0-D-Phe1-Tyr3-octreotide) and two dedicated, commercially available PET/CT scanners (Biograph Horyzon 16, Siemens Healthcare GmbH, Erlangen, Germany, and GE Discovery 690, GE Healthcare, Chicago, IL, USA).

A 110 MBq dose of 68Ga-DOTATOC was administered intravenously to each patient. After 50 min, we performed the PET/CT scan, with the patient in supine position. The PET scan of the skull was acquired in three-dimensional list mode for 15 min. The CT component was of diagnostic quality for both scanners (120 kV; 250 mAs; 3 mm slice). 

The PET reconstruction settings were subjected to CT-based attenuation correction, using an ordered subset expectation maximization (OSEM) algorithm (for the Biograph Horyzon: method = TrueX+TOF, iterations = 12, subsets = 10, image matrix = 512, voxel size = FWHM 2 mm; for the GE Discovery 690: method = Vue Point FX, iterations = 5, subsets = 16, image matrix = 256, voxel size = FWHM 3.2 mm). 

**Figure 1:**
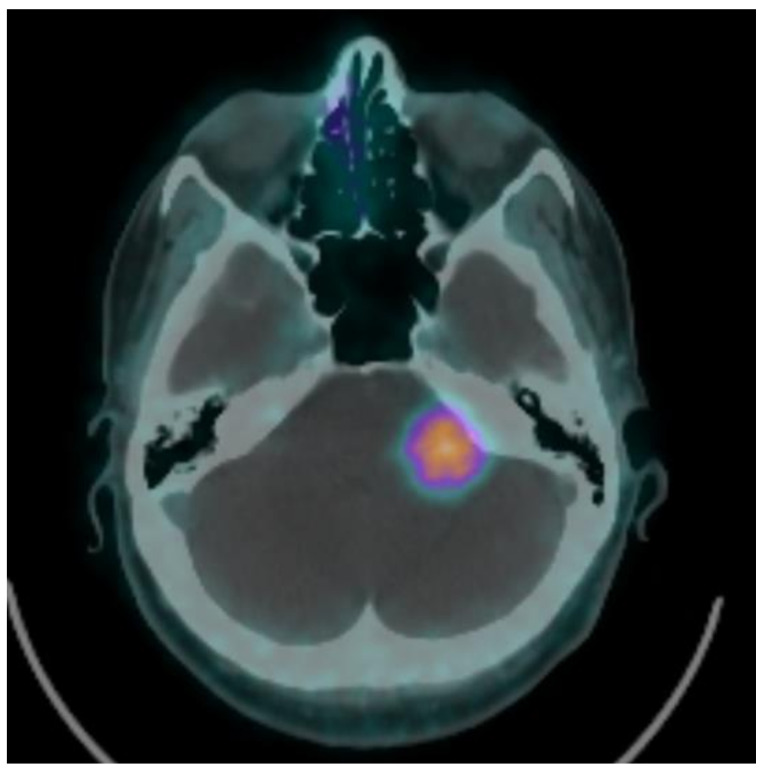
^68^Ga-DOTATOC positron emission tomography (PET)/CT allows differential diagnosis with caustic schwannoma. Standardized uptake value (SUV): 7.1.

A Gaussian filter of 2 mm in full width at half maximum was then applied to all images after reconstruction. The PET slice thickness was 2 mm (Figure 1).

### 2.2. Planning and Treatment

**Figure 2:**
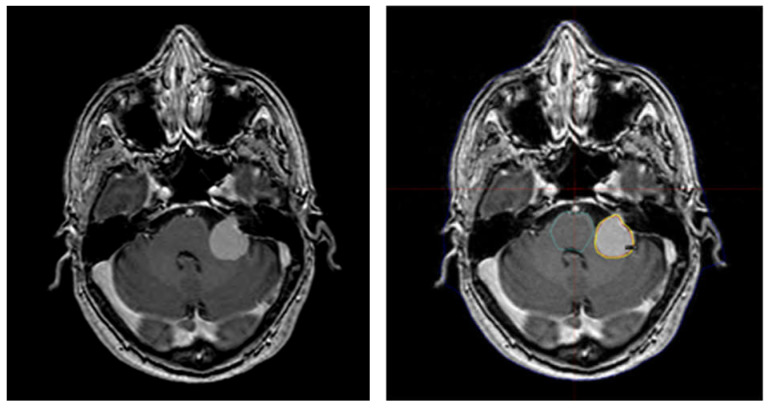
Left: MRI showing the enhancement post-gadolinium administration. Right: MRI of the brain to contour the tumor on 1.5T clinical scanners (Philips Achiva, Best, The Netherlands) used in the planning stage with gamma plan station. Images were transferred to the gamma plan working station. The tumor showed a volume of 5514 cm^3^. Treatment was delivered by the Leksell Gamma Knife Icon^TM^ (Elekta, Stockholm, Sweden) as a single dose with 13 Gy marginal dose, 50% isodose.

The case series presented includes patients referred to our Gamma Knife center after an accidental diagnosis of meningioma. The indication to treat those lesions with Gamma Knife derived from a multidisciplinary meeting with patient and family, neurosurgeons, and radiotherapists. A physicist joined the Gamma Knife team in the planning phase, in which an MRI of the brain was used to contour the tumor on 1.5T clinical scanners (Philips Achiva, Best, The Netherlands) that included T1W 3D-TFE (turbo field echo) TR/TE 7.5/3.4, slice thickness = 1 mm, FA (flip angle) = 8°, TFE factor = 240, FOV = 240 × 240 (Figure 2). 

Data sets were also used to establish the tumor volume. The MRI set was used to precisely define tumor boundaries with cerebrospinal fluid (CSF), venous sinuses, and brain parenchyma. We divided the patient population into two groups: group A, patients who underwent pre- and post-Gamma Knife ^68^Ga-DOTATOC PET/CT to analyze the possible post-Gamma Knife SUV modifications, and group B who underwent only pre-Gamma Knife ^68^Ga-DOTATOC PET/CT to confirm the diagnosis of meningioma. The Gamma Knife radiation dose was delivered as single fraction in all but two patients in group A, who received 5 fractions. At the gamma plan (Leksell GammaPlan^®^, Elekta, Crawley, UK), critical structures were identified, and the dose was established: single fraction ranged from 12.5 to 14 Gy, while the two hypofractionated treatments consisted of 5 fractions, one 4, and the other 5 Gy, 50% isodose, 20 and 25 Gy, respectively, as maximal dose.

Treatment was delivered by the Leksell Gamma Knife Icon^TM^ (Elekta, Stockholm, Sweden), using up to 192 low-intensity radiation beams from cobalt-60 sources converging with high accuracy on the target. It is deliverable as a Leksell frame-based or frameless procedure with a thermoplastic mask, according to target volume and patient clues. The device elaborates data, from the image guidance system, consisting of a stereotactic MRI and an integrated stereotactic cone-beam CT, and determines coordinates in 3D after co-registration with MR images, as in the frameless treatments. The 192 cobalt-60 beams were not coplanar, so some or all of them could be adjusted to compensate for smaller patient movements, particularly during frameless procedures, achieving a perfect 6D positioning. The patient was monitored continuously during treatment with a movement resolution of 0.15 mm, automatically turning off the beam delivery when it was intolerable. When a target volume close to a highly functional structure is planned to be treated, the shape of a single shot may be adapted to obtain effective treatment and spare organs at risk. 

### 2.3. Statistical Analysis

Data were collected manually (Table 1). 

The descriptive statistics included median (range) for continuous variables (e.g., age, SUV level), and number (percentage) for categorical variables (e.g., sex) were calculated. The correlation between continuous variables was assessed using the Pearson correlation test. Statistical analysis was conducted using the R package.

## 3. Results

Twenty patients were included in this study, 10 females and 10 males, with a median age of 52 years (range 33–80). The average tumor diameter was 3.68 cm (range 0.12–22.26 cm), and the median pre-radiotherapy maximum SUV value was 11 (range 2.3–92). Of the 20 patients, 18 received the treatment in a single fraction. The mean marginal dose was 13 Gy (range 12.5–16) (Table 2).

**Figure 3:**
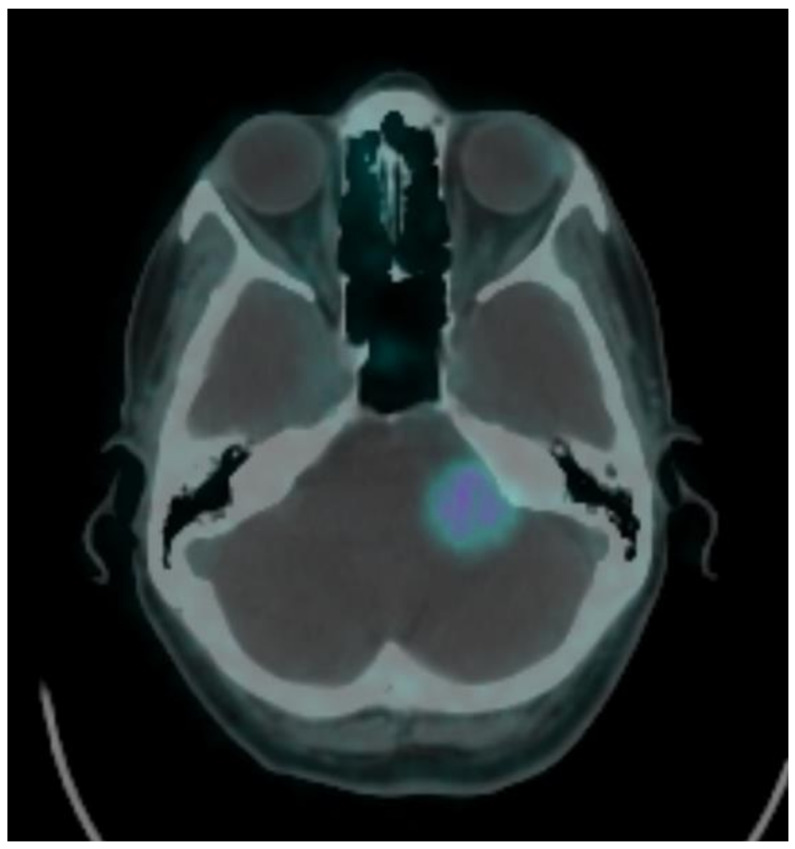
^68^Ga-DOTATOC PET/CT follow up at 6 months showing SUV intensity and reduction of the lesion from 7.1 to 4.1 cm.

In two patients, the tumor diameter was larger than 20 cm. In one of these patients, the marginal dose was 20 Gy in 5 fractions, while in the other patient it was 25 Gy in 5 fractions. The average relative percentage change between SUVs at baseline and follow-up was −6%, ranging from −41% to 56%. The SUV was reduced in seven out of 12 patients (58%) (Figure 3), stable in two out of 12 (17%), and increased in three out of 12 (25%), suggesting a biological response of the tumor to the Gamma Knife treatment in most of the cases. 

**Figure 4:**
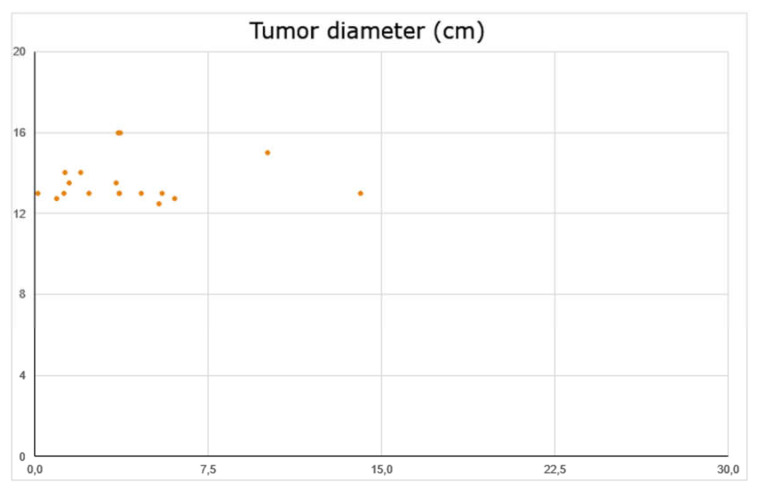
Tumor diameter versus minimal dose to the prescribed isodose level.

There was no statistical correlation between the tumor diameter or volume and the SUV pre- or post-radiotherapy or the relative percentage changes between SUVs at baseline and follow up (Figure 4). 

## 4. Discussion

The use of ^68^Ga-DOTATOC PET/CT imaging has been reported in only a few papers, highlighting its utility in distinguishing meningiomas and metastases. Due to the expression of SSTR-2 receptors, meningiomas show a much higher uptake compared with dural metastases. A visual scoring system has been devised to further validate such a technique and differentiate better meningiomas from other tumors expressing SSTR-2 (breast cancer, thyroid cancer, Merkel cell carcinoma, lymphoma). Purandare et al. reported that three quarters of the patients with meningeal lesions showed intense tracer uptake, equal to that of the spleen (visual score 3), whereas all metastatic lesions showed a lower visual score (visual score < 3).

In the literature, only a few pre-radiosurgical gallium–PET studies, CT–PET or MRI–PET based, have been reported. In the present study, we suggest using ^68^Ga-DOTATOC PET/CT as a valuable tool both during the pre-radiosurgical assessment, to confirm the diagnosis and the need for radiosurgery, and in the setting of patients who have not been surgically treated thus without histologic examination of the lesion target of the Gamma Knife treatment. According to Gehler et al. [17], ^68^Ga-DOTATOC-PET offers detailed information on tumor characteristics during Intensity-modulated radiation therapy (IMRT) planning. He presented a case series of 26 patients, in which ^68^Ga-DOTATOC-PET imaging allowed a better assessment of the tumor extension in 17 of them (65%); the PET GTV presented lower values if compared to CT/MRI in 10 out of 26 patients (38%), higher in 13 (50%), and equal in three out of 26 patients (12%). Other authors investigated the utility of ^68^Ga-DOTATOC-PET in fractionated stereotactic radiation therapy (FSRT) planning. They reported a significant reduction in the size of the lesion in 10–38% of cases [18] and better definition of the target in 73% of patients [13,19].

The utility of ^68^Ga-DOTATOC-PET has also been reported in the setting of photon and proton treatment, again showing a reduction of the target volume in most patients [20]. The identification of the CTV is related to the microscopic extension of the tumor, which can be determined by using molecular imaging, mainly PET–CT studies. Nevertheless, PET–CT imaging does not allow accurate detection of all the clonogenic tumor cells, and this technical limitation affects the accuracy of the radiosurgical planning. To be noted, ^68^Ga-DOTATOC-PET-MRI has also been reported to provide useful information at the level of organ at risk (OAR). Some authors have stated that ^68^Ga-DOTATOC-MRI planning allowed to treat tumor regions that would not have been treated otherwise in 54% of patients, in a small series of 11 cases [21]. Considering the exact match of the planning target volume (PTV) with gross total volume (GTV), ^68^Ga-DOTATOC-PET imaging offers fine target boundary identification, which is of the utmost importance in radiosurgery, Gamma Knife, stereotactic radio surgery (SRS), and FSRT, in which the target is usually closer to the OARs, without safety edges. Although ^68^Ga-DOTATOC-PET has been reported to reduce the target volume, which is important with CyberKnife, whereas, in our experience, ^68^Ga-DOTATOC-PET offers the opposite possibility when using Gamma Knife, that is, to increase the target volume. As a matter of fact, Gamma Knife does not need any safe tissue near the target because the dose decreases immediately at the target’s edge. In our series, we could expand the target, with the help of the information provided by the ^68^Ga-DOTATOC-PET, in two patients affected by parasagittal meningiomas with infiltration of the superior sagittal sinus, not visible at the MRI but documented by the PET scan. Furthermore, the new Gamma Knife ICON allows fractionated treatments, from which larger lesions, often characterized by infiltration of sinuses or eloquent skull base structures, may benefit. In this setting, ^68^Ga-DOTATOC-PET imaging provides useful information about the planning, as well as a functional diagnosis for primary radiosurgery. In our practice, PET–CT images are merged with MR images, improving windowing and tumor definition. In our experience and in agreement with other authors, for tumors of the skull base and especially of the sellar and parasellar regions, PET imaging is burdened by the significant uptake of the pituitary gland, which makes it challenging to clearly distinguish the tumor and the unaffected tissue [20]. An important consideration is that the patient population mainly included asymptomatic patients, affected by tumors affecting eloquent structures, detected during MRI assessment for general investigation (because of dizziness, headache etc.). In this setting, ^68^Ga-DOTATOC-PET, alone or merged with MRI imaging, offers concrete advantages in the planning, with consequent clinical benefits. To the best of our knowledge, this is the first time ^68^Ga-DOTATOC-PET has been reported as an aid in the diagnosis definition without histological examination, together with a ^68^Ga-DOTATOC-PET follow up after single fraction or hypofractionated Gamma Knife treatment. According to our results, a pre-radiosurgery ^68^Ga-DOTATOC-PET was performed on all patients, and 12 underwent another one during follow up. The SUV was reduced in seven out of 12 patients, stable in two out of 12, and increased in three out of 12, suggesting a biological response of the tumor to the Gamma Knife treatment in most of the cases. Of notice, the two patients who underwent hypofractionated treatment showed a reduction in the SUV like the single fraction group, suggesting effective therapeutic effects of the Gamma Knife also in the hypofractionated setting. The SUV stability after Gamma Knife in two patients, as well as the increased SUV, should be considered a further demonstration of the efficacy of the treatment, in agreement with the existing literature [22,23,24,25]. The increase in SUV was probably related to the tumor’s histology, which is not known due to the small size of the tumor and, thus, the absence of surgery in these asymptomatic patients. It is reasonable to expect a late favorable response by those tumors, which will be evaluated in a late ^68^Ga-DOTATOC-PET follow up. It should be noted that none of the tumors showed any growth in size. The post-Gamma Knife ^68^Ga-DOTATOC-PETs were performed at random time intervals to evaluate possible early or late modifications of the SUV, and our results have proven that there is no clear correlation with the time interval, given that there was a random distribution of the SUV modification. The long-term SUV modifications, the associated radiological characteristics, and patient outcome require further investigations and a longer follow up. Despite this, we highlight that the aim of the paper was not to investigate only the efficacy of single-fraction or hypofractionated Gamma Knife treatment but rather to provide initial data about the utility of the use of ^68^Ga-DOTATOC-PET in the assessment of the diagnosis of meningioma without histological examination and how this imaging method can also offer further useful information during follow up.

## 5. Conclusions

The present observational study provides promising information about the use of ^68^Ga-DOTATOC-PET to assess the diagnosis of meningioma for primary radiosurgery and as a valuable tool during the follow up after single fraction or hypofractionated gamma-knife treatment. Further investigation is needed on a larger cohort of patients and with longer follow up.

## Figures and Tables

**Table 1:** Patient population characteristics and summary of the tumor size, SUV values, and Gamma Knife ICON dose administration.

Number of Patient	Years	Sex	Location	Tumor Diameter (cm)	Tumor Volume (cm^3^)	Gy Gamma Knife	SUV Pre-Gamma Knife	PET Time from Gamma Knife (Months)	SUV Post-Gamma Knife	DeltaSUV	D_Marg
1	36	male	right fronto-orbital	3.679	208.476808	13	10.2	19	13.1	28%	13.0
2	56	female	left cerebellar	1.310	9.41200765	14	3.5	18	2.6	−26%	14.0
3	63	male	left petroclival	4.593	405.655829	13	2.3	14	2.3	0%	13.0
4	36	female	right tentorial	5.4	659.24928	12.5	53	11	35.7	−33%	12.5
5	33	male	right parasagittal	22.265	46210.0548	5	13	8	12.5	−4%	
6	47	male	left parasagittal	1.494	13.9611173	13.5	31.6	9	44.6	41%	13.5
7	72	female	left cerebellopontine angle	5.514	701.889378	13	7.2	6	4.3	−40%	13.0
8	58	female	olfactory groove	6.046	925.279229	13	25.8	5	15.1	−41%	12.8
9	58	female	left cavernous sinus	0.931	3.37844947	12.5	11.5	1	10.6	−8%	12.8
10	70	male	right clinoidal	3.517	182.131678	13.5	24	3	24	0%	13.5
11	50	female	left sphenopetrocavernous	19.8	32498.5478	4	20.8	15	18.4	−12%	
12	52	male	right posterior petrous bone	2	33.4933333	14	68.6				14.0
13	55	male	right parasagittal	10.078	4285.4008	15	7.1				15.0
14	80	male	left parietal	3.608	196.638237	16	9.8				16.0
15	80	male	right tentorial	3.712	214.137283	13.5	7.5				16.0
16	65	female	left parieto-occipital	14.1	11736.1519	13	9				13.0
16	52	female	left cerebellar	2.35	54.3340367	13	2.9				13.0
18	52	female	right posterior petrous bone	1.284	8.86264709	13	92				13.0
19	51	female	right tentorial	0.118	0.00687883	13	16.3				13.0
20	47	male	left frontal	3.684	209.327964	13	10.4		16.2	56%	13.0

**Table 2:** Focus on pre- and post-Gamma Knife SUV mean values, mean age, tumor diameter distribution.

		Average Age	Minimum	Maximum
Female	10	54.29	33	80
Male	10			
Total	20			
		Average SUV_pre	Minimum SUV_pre	Maxium SUV_pre
		21.325	2.3	92
		Average D_marg	Minimum D_marg	Maxium D_marg
		14.45 Gy	12.5 Gy	25 Gy
		Average Tumor diameter (cm^3^)	Minimum Tumor diameter (cm^3^)	Maxium Tumor diameter (cm^3^)
		5.78	0.118	22.27
